# Ultra-early follow-up computed tomography detects hematoma expansion in intracerebral hemorrhage with high accuracy

**DOI:** 10.3389/fneur.2025.1701854

**Published:** 2026-01-29

**Authors:** Jawed Nawabi, Kira Koch, Andrea Dell’Orco, Andrea Morotti, Georg Bohner, Michael Scheel, Eugen Schwabauer, Joachim E. Weber, Kersten Villringer, Justus F. Kleine, Mike P. Wattjes, Horst Urbach, Heinrich Audebert, Frieder Schlunk

**Affiliations:** 1 Department of Neuroradiology, Charité–Universitätsmedizin Berlin, Corporate Member of Freie Universität Berlin and Humboldt-Universität zu Berlin, Berlin, Germany; 2 Berlin Institute of Health, Berlin, Germany; 3 Neurology Unit, Department of Clinical and Experimental Sciences, University of Brescia, Brescia, Italy; 4 Department of Neurology, Charité–Universitätsmedizin Berlin, Freie Universität Berlin, Humboldt-Universität zu Berlin, Berlin, Germany; 5 Center for Stroke Research Berlin, Charité–Universitätsmedizin Berlin, Corporate Member of Freie Universität Berlin and Humboldt-Universität zu Berlin, Berlin, Germany; 6 Department of Neuroradiology, Medical Center–University of Freiburg, Freiburg im Breisgau, Germany

**Keywords:** computed tomography, hematoma expansion (HE), intracerebal hemorrhage, mobile stroke unit, stroke

## Abstract

**Background and objectives:**

This study investigates whether ultra-early follow-up imaging can reliably identify patients at risk for hematoma expansion (HE) in acute intracerebral hemorrhage (ICH).

**Methods:**

In this multicenter study, we analyzed data from patients with primary ICH who underwent at least two non-contrast cranial computed tomography (NCCT) scans within 7 days of symptom onset or last known well. To define the optimal time window, hematoma growth dynamics were retrospectively assessed in a large cohort (*n* = 1,663). Based on these findings, a prospective sub-study included patients with repeated imaging within 200 min (*n* = 46). HE was defined as >6 mL or >33% volume increase between the admission and the second follow-up scan. The diagnostic performance of early volume increase was evaluated using receiver operating characteristic (ROC) analysis.

**Results:**

The highest proportion of patients with active hemorrhage was detected within the first 200 min in the initial phase of this study. In the prospective sub-study, percentage volume increase between admission and early follow-up imaging demonstrated excellent diagnostic performance for HE (AUC = 0.819). At an optimized cutoff, the model yielded a sensitivity of 0.885 and a positive predictive value (PPV) of 74%. Among patients with early expansion already visible at follow-up, 50% showed further volume increase on the final scan. A separate analysis limited to follow-up imaging within the first 120 min after symptom onset (*n* = 27) revealed a higher diagnostic accuracy, with an AUC of 0.846 (sensitivity 0.857; PPV 71%).

**Conclusion:**

ICH evolves rapidly in the first hours after onset. Follow-up imaging within the first 200 min can diagnose hematoma growth with high sensitivity and good accuracy. However, the inability to distinguish between ongoing and completed expansion underscores the need for additional imaging or clinical markers to support clinical decisions.

## Introduction

Randomized clinical trials (RCT) aiming to limit hematoma expansion (HE) in acute intracerebral hemorrhage (ICH) have so far failed to prove a convincing therapeutic effect (1–3). One major reason may be that the diagnostic accuracy of imaging-based predictors of HE used to select treatment-eligible patients is limited (4). Therefore, there is an unmet medical need for a diagnostic tool capable of identifying patients at high risk for further hematoma enlargement, to guide both RCTs and clinical treatment in the future.

Although the exact dynamics of hematoma growth in ICH remain unclear, clinical data suggest that HE in acute ICH occurs in the early phase, as patients presenting soon after symptom onset are more likely to experience expansion, consistent with the “stroke like” onset of symptoms (5, 6). However, significant HE is observed in patients up to 6 h after symptom onset and in a minority during the later course (7). HE represents a promising target for acute ICH treatment, since even a small increase in hematoma volume worsens functional outcomes and mortality, and because it is potentially modifiable (8).

Imaging markers, such as the computed tomography angiography (CTA) spot sign and several non-contrast cranial computed tomography scans (NCCT) features of ICH, are associated with HE (9, 10). While these markers are useful for understanding the disease biology and presumably indicating active bleeding, their utility for rapid stratification of HE risk in the acute clinical setting remains limited. In fact, two RCTs that used the CTA spot sign to select patients with ICH for treatment with recombinant factor VIIa, were terminated prematurely (4). The main problem of CTA spot sign assessment may be that it captures only a contrast leak at the time of examination and therefore cannot reliably rule out HE beyond the acquisition window. Accurate prediction of HE in ICH remains a critical unmet need in acute care, both to support clinical providers and to guide the design of future RCTs.

This study hypothesized that ultra-early follow-up up NCCT imaging can be used to diagnose HE in ICH with high accuracy and conducted a retrospective as well as a prospective multicenter study, using repetitive NCCT imaging in the early phase of acute ICH.

## Methods

### Study population

Deidentified data will be made available on reasonable request through email to the corresponding author, following review and approval of a research proposal by the trial executive committee along with a signed data access agreement.

For this study, we retrospectively identified all patients diagnosed with ICH between January 2014 and September 2022 at Charité Universitätsmedizin Berlin. In addition, data were obtained from three other large hospitals in Berlin that entered their clinical and imaging data into the prospective “Berlin–specific acute treatment in ischemic and hemorrhagic stroke with long-term outcome” (B-SPATIAL) registry. B-SPATIAL was implemented as a quality ascertainment registry collecting data of all acute stroke patients who arrived within 6 h of symptom onset to one of 15 Berlin hospitals with a Stroke Unit between 2016 and 2021. All of these hospitals also receive patients from mobile stroke units equipped with onboard CT scanners, enabling ultra-early imaging to be performed directly in the field prior to hospital arrival. Adults (>18 years) with spontaneous supratentorial or infratentorial ICH were included if they had undergone both an initial non-contrast CT (NCCT) and follow-up NCCT within 7 days of admission. Patients transferred from external hospitals were eligible if the digital imaging and communications in medicine (DICOM) data of their initial NCCT were available. Individuals receiving oral anticoagulant therapy were not excluded.

Exclusion criteria included ICH secondary to brain tumors, vascular malformations, head trauma, or hemorrhagic transformation of ischemic infarction, as well as primary intraventricular hemorrhage. Patients who underwent surgical interventions such as decompressive craniectomy or hematoma evacuation prior to follow-up imaging were also excluded.

This study was approved by the Ethics Committee of Charité Universitätsmedizin Berlin (approval numbers EA4/009/20 and EA4/109/15). The waiver of informed consent was based on the specific Berlin Hospital Legislation (Berliner Krankenhausgesetz), which permits the use of routine clinical data for research purposes within the same hospital and data from quality ascertainment registries in case of hospital collaborations. Data from B-SPATIAL registry were used after an opt-out procedure, following approval by the institutional Data Protection Representatives of the contributing hospitals (11). All procedures were conducted in compliance with the Declaration of Helsinki.

### Study design

**Figure 1:**
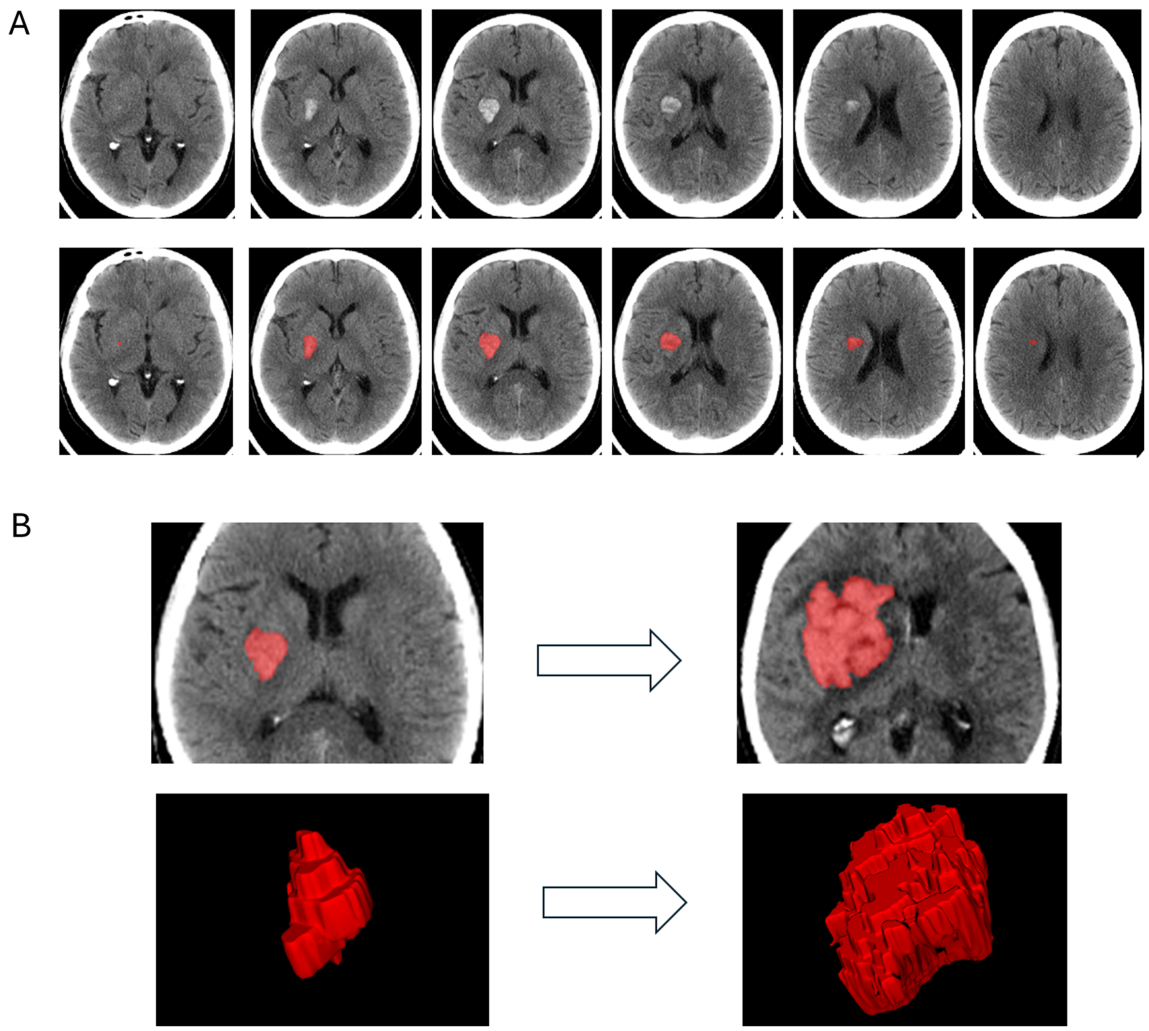
**(A)** Segmentation process of a deep intracerebral hemorrhage (ICH) on 5 mm non-contrast CT (NCCT) slices using ITK-SNAP. Upper row: Axial NCCT images showing a right-sided deep basal ganglia hematoma. Lower row: Corresponding manual segmentation of the hematoma, illustrating the delineation process. **(B)** Longitudinal imaging of the same intracerebral hemorrhage (ICH) as in **(A)**. Upper row: Axial NCCT slices at admission (left) and at follow-up (right) with overlaid manual segmentations, indicating marked hematoma expansion. Lower row: Corresponding 3D reconstructions of the complete manual segmentations (left: admission, right: follow-up).

**Figure 2:**
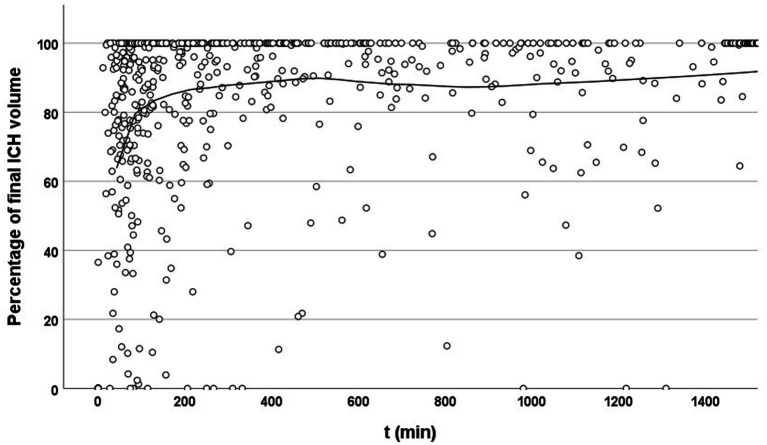
Temporal evolution of hematoma volume relative to final size. Scatter plot showing the percentage of final intracerebral hemorrhage (ICH) volume at each time point after symptom onset or last known well (*t*, in minutes). Each dot represents an individual imaging time point. The solid line indicates a locally estimated scatterplot smoothing (LOESS) curve. A clear upward trend is visible within the early time window (<200 min), suggesting that imaging during this period often captures substantial hematoma growth. Beyond this window, measured volumes increasingly approach 100%, reflecting completed or stabilized expansion.

This study was divided into two phases. In the first phase, the entire multicenter dataset was analyzed to identify an appropriate time window for the subsequent sub-study. We included patients with at least two imaging sessions (admission and follow-up) within the first 7 days after symptom onset or last known well, respectively. In this phase, the last imaging session (between 24 h and 7 days after symptom onset) was used for measuring the final hematoma volume (see Figure 1). Hematoma volumes from prior imaging session(s) were expressed as a percentage of this final volume and plotted on a growth curve. If a third imaging session occurred later than 7 days post-admission and the second imaging session was between 24 h and 7 days, the second imaging session was defined as the final hematoma volume, and only the admission imaging volume was expressed as a percentage of this reference. The time of symptom onset or last known well, respectively, was defined as 0 min and 0 mL on the plot (12) (see Figure 2).

**Figure 3:**
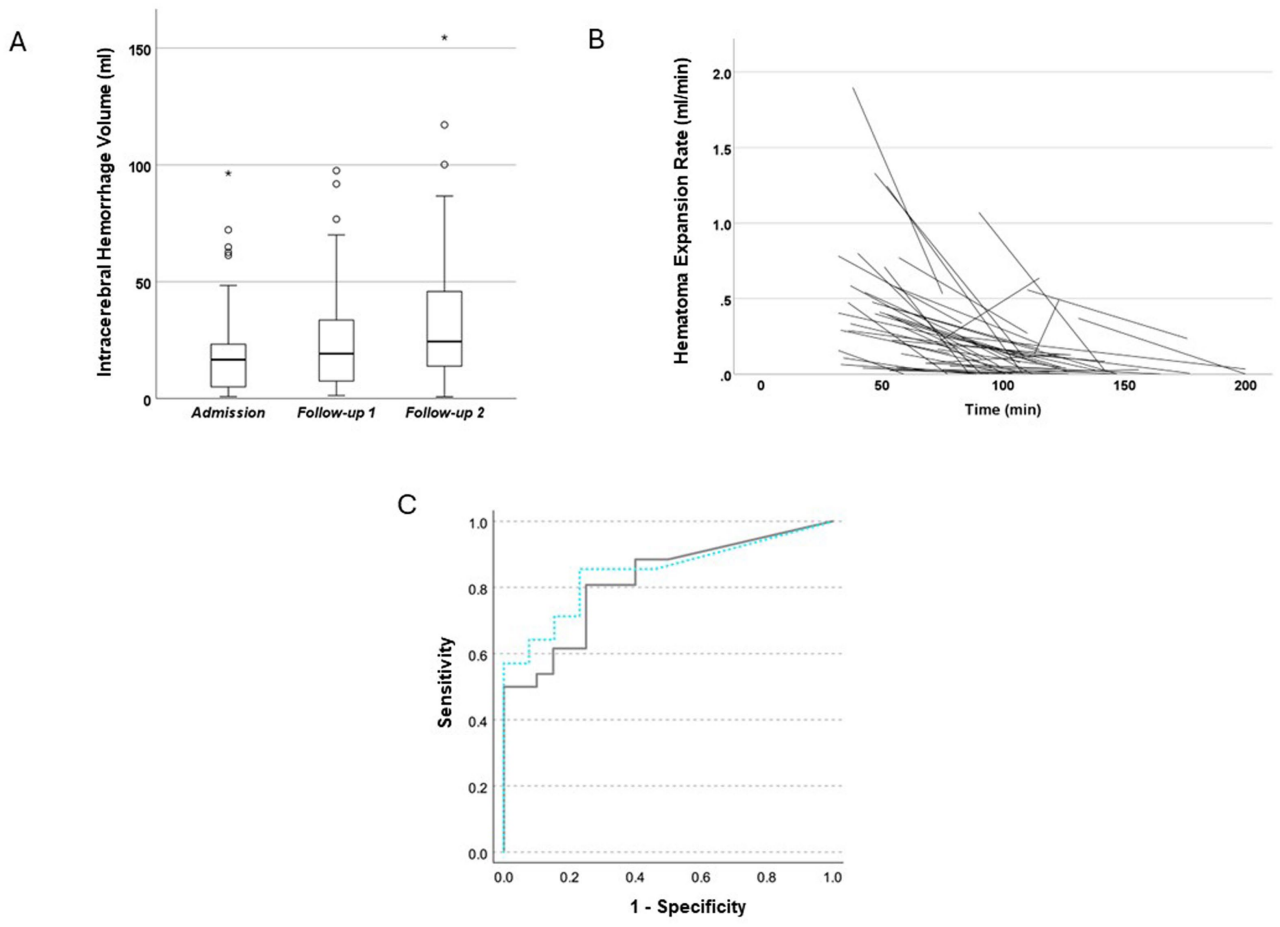
**(A)** Distribution of intracerebral hemorrhage volumes at three time points: Admission, follow-up 1, and follow-up 2. Hematoma volume increased significantly over time. Boxes represent the interquartile range (IQR), horizontal lines indicate the median, whiskers show 1.5 × IQR, circles indicate mild outliers, and asterisks denote extreme outliers. Data shown for patients with prospectively acquired follow-up NCCT scans within 200 min from symptom onset. **(B)** Speed of hematoma expansion in patients with the first follow-up CT within 200 min after symptom onset. The figure shows that in nearly all cases, hematoma growth speed decreases over time, starting as early as approximately 35 min after symptom onset. **(C)** Receiver operating characteristic (ROC) curve illustrating the diagnostic performance of percentage volume increase between admission and follow-up imaging in detecting hematoma expansion or stability. The ROC for the 200-min time window is shown as a solid gray line (AUC = 0.82; sensitivity = 0.889; specificity = 0.526; positive predictive value [PPV] = 74.2%; negative predictive value [NPV] = 80.0%), and for comparison the ROC for the 120-min time window is shown as a dashed turquoise line (AUC = 0.846; sensitivity = 0.857; specificity = 0.462; PPV = 71%; NPV = 80%).

After defining the appropriate time window for the sub-study in the first phase—based on the steepest segment of the hematoma growth curve shown in Figure 2—we included all patients who had admission and repeated imaging within the first 200 min after symptom onset or last known well, and who underwent a third imaging session within 7 days of admission. In a further analysis, this study investigated a narrower time window of 120 min, during which hematoma growth appeared particularly dynamic. The speed of hematoma expansion (mL/min) was calculated for each patient. The ICH volume on admission CT was divided by the time interval from symptom onset or last known well to the admission scan. For follow-up CT, the difference in hematoma volume between follow-up and admission was divided by the time interval between the two scans (Figure 3B).

This subset, drawn from all participating centers, was analyzed separately and prospectively as part of the Berlin prehospital or usual care delivery in stroke (B_PROUD) study, in which an ultra-early follow-up NCCT was routinely performed upon hospital arrival after a prehospital NCCT had already been acquired by the mobile stroke unit (11, 13).

### Image acquisitions and analysis

NCCT scans were performed on an 80 or 320 slice scanner with the following imaging parameters: incremental acquisition at 120 kV, 280 mA, 1 mm, and 5 mm slice reconstruction. Prehospital scans on mobile stroke units were developed on a portable 8-slice CT scanner.

A senior neuroradiologist reviewed each case, confirming hematoma location and the presence of intraventricular hemorrhage. Hematoma volumes were quantified on axial CT slices (5 mm thickness) using a semi-automated segmentation tool (ITK-SNAP, version 3.8.0). Measurements were executed by multiple experienced stroke imaging readers, all blinded to patient demographics and outcomes. NCCT scans were examined in a random order. This method was previously validated in a study by our group, demonstrating excellent intra- and interrater reliability (14).

### Statistical analysis

Data analysis was performed using IBM SPSS Statistics, version 29.0.0.0 (241). Normally distributed continuous variables are reported as mean and standard deviation (SD), whereas non-normally distributed variables are expressed as median and interquartile range (IQR). Categorical variables are summarized as absolute counts and percentages. Baseline demographic, clinical, and radiological characteristics were described, with no formal statistical comparisons between patient sub-groups. Changes in hematoma volume (prospective sub-study; Figure 3A) across multiple imaging time points were conducted using the Friedman test for repeated measures. Pairwise comparisons between time points were performed using Wilcoxon signed-rank tests, with Bonferroni correction applied to adjust for multiple testing. Receiver operating characteristic (ROC) analysis was applied to examine the ability of the percentage hematoma volume increase between admission and follow-up imaging to discriminate HE from stability. HE was defined as a relative increase of more than 6 mL or more than 33% between baseline NCCT and final follow-up imaging. Areas under the curve (AUCs) were calculated using a non-parametric trapezoidal method. ROC analyses were conducted separately for two time windows (≤120 min and ≤200 min from symptom onset or last known well). Because the 120-min cohort was a subset of the 200-min cohort and differed in sample size, ROC curves were compared descriptively and visually only. No formal statistical comparison between AUCs (e.g., DeLong test) was performed, as such methods require identical patient samples.

## Results

### Study participants and imaging data

Between January 2014 and September 2022, the database recorded 1,663 patients diagnosed with ICH. The median age was 73 (61–81 years), and 730 (44%) were female. Hypertension (*n* = 1,356, 83%) and diabetes (*n* = 296, 18%) were common comorbidities. Patients were recruited primarily from Charité Universitätsmedizin Berlin, the main study center (*n* = 718). ICHs were classified as supratentorial deep in 299 patients (42%), supratentorial lobar in 304 patients (43%), and infratentorial in 111 patients (16%). Further baseline demographics and clinical characteristics are shown in detail in Table 1. For the final analysis, patients who underwent an initial NCCT and follow-up imaging within 7 days (*n* = 1,086) were included. A prospective sub-study was conducted, selecting 46 subjects who received an ultra-early follow-up NCCT within the first 200 min and who underwent a third imaging session within 7 days of admission.

**Table 1:** Baseline demographic, clinical, and radiological characteristics in patients with acute intracerebral hemorrhage (ICH).

Variable	Total (*N* = 1,663)
Age, median (IQR), y	73 (61–81)
Female sex, No. (%)	730 (44)
Hypertension, No. (%)	1,356 (83)
Diabetes, No. (%)	296 (18)
Antiplatelet therapy^a^, No. (%)	170 (26)
Anticoagulation therapy^a^, No. (%)	202 (30)
Glasgow Coma Scale score, median (IQR)	12 (8–15)
NIHSS admission, median (IQR)	11 (5–17)
mRS discharge, median (IQR)	5 (4–6)
Bleeding location^a^, No. (%)	
Deep	299 (42)
Lobar	304 (43)
Cerebellar	75 (11)
Brainstem	36 (5)
Baseline ICH volume, median (IQR), mL	18 (6–45)
Time to admission CT scan, median (IQR), min	149 (75–410)
Time to first follow-up CT scan, median (IQR), min	1,270 (538–2,191)
Time to final CT scan, median (IQR), min	3,679 (1,836–7,078)

a Data available only from the largest contributing center (*n* = 718).

NIHSS, National Institutes of Health Stroke Scale; mRS, Modified Rankin Scale; IQR, Interquartile range.

### Hematoma expansion–descriptive analysis

Most patients reached approximately 60–80% of their final ICH volume within 100 min of symptom onset. The growth curve then began to flatten, although substantial expansion continued over the next 100 min. Beyond this period, hematoma growth approached a plateau over subsequent hours (Figure 2).

When analyzing only patients who had not yet reached their final hematoma at time of their admission or follow-up NCCT scan, the slope of the curve remained similar, with hematoma volumes reaching approximately 70% at 100 min. These findings highlight the considerable number of patients who could benefit from effective treatment. Based on our findings, we defined the time window for our preplanned sub-study as 0–200 min after symptom onset or last known well.

### Prospective sub-study of patients undergoing ultra-early NCCT follow-up

About 46 patients underwent prehospital NCCT in the mobile stroke unit, followed by an ultra-early follow-up NCCT upon hospital arrival, which was routinely performed as part of the preplanned B_PROUD protocol, and additional imaging within 7 days to determine the final hematoma volume. Median time from symptom onset or last known well to first NCCT was 54 min (IQR 40–74), 115 min (93–131) to the first follow-up, and 1,164 min (782–1,668) to the final follow-up, respectively. Median hematoma volume at admission was 17 mL (IQR: 5–24 mL) in the first scan, 19 mL (IQR: 7–34 mL) at follow-up, and 24 mL (IQR: 13–48 mL) at final imaging (Figure 3A). Hematoma volume increased significantly over time [Friedman test: χ^2^(2) = 29.391, *p* < 0.001]. Pairwise comparisons using Wilcoxon signed-rank tests with Bonferroni correction revealed significant differences between the different time points (adjusted *p* = 0.02–<0.001). Approximately, 57% of patients suffered from HE >6 mL and/or 33% measured on final imaging scans. The speed of growth decreased rapidly over time and fell below 0.5 mL/min after 75 min in almost all cases (Figure 3B). Using ROC analysis, the percentage volume increase between admission and follow-up imaging proved to be an excellent diagnostic tool for identifying HE or stability (AUC 0.82; sensitivity 0.889, specificity 0.526; Figure 3C). At the optimal cut-off point, the PPV was 74.2%, and the NPV was 80.0%. Of those patients with early expansion visible at first follow-up, 50% exhibited further volume increase on the final scan, whereas the remaining patients had already reached their final volume by that time. Substantial further hematoma growth was observed in only a subset of patients (e.g., with 46% of those with initial HE showing an additional increase >1 mL and 37% showing an increase of >3 mL). When the time window was narrowed to 120 min (i.e., when HE appeared particularly dynamic), the accuracy of the study model improved when visually comparing the ROC curves (AUC = 0.846; sensitivity = 0.857; specificity = 0.462; PPV 71%; NPV 80%; *n* = 27; Figure 3C).

## Discussion

In this retrospective multicenter study, the first 200 min after ICH represent the critical time window with the highest proportion of patients experiencing ongoing hematoma growth. Within this time window, follow-up NCCT accurately detected hematoma expansion or stability, regardless of the time interval between admission and follow-up NCCT.

Our data demonstrate that the greatest dynamics of hematoma expansion occur within the first 200 min after ICH onset. Notably, growth speed declined markedly as early as 35 min after presumed bleeding onset in almost all cases. While similar observations have been made by other groups, our dataset—prospectively collected with exceptionally prompt imaging from mobile stroke units—represents, to our knowledge, the earliest dataset providing insight into the initial phase of hematoma expansion (12, 15).

Trials of acute ICH treatments aimed at reducing hematoma growth have largely failed to demonstrate clinical benefit, potentially due to inadequate identification of patients at high risk for HE. Two randomized trials that used the CTA spot sign—present in approximately one-quarter of patients with acute ICH and associated with HE and poor outcomes—as a treatment trigger yielded negative results (4). By contrast, an ultra-early follow-up NCCT scan, is readily implementable in clinical practice and, in our study, accurately identified both HE and stability. The diagnostic performance might be further enhanced when combined with established imaging markers such as the CTA spot sign or non-contrast CCT features, a strategy that warrants exploration in future trials.

However, it is important to acknowledge that only a subset of patients with HE exhibited further growth after ultra-early follow-up imaging. This aligns with previous studies, including secondary analyses from the SPOTLIGHT and STOP-MSU trials, suggesting that many hematomas expand and cease very early (3, 12, 15, 16). In our cohort, both admission and follow-up imaging were performed at even earlier time points, which may explain the comparatively higher rate of hematoma expansion observed. Taken together, these findings emphasize that this approach may not be suitable as a treatment trigger in randomized clinical trials, where timely identification of truly active bleeding is critical. Instead, a treatment strategy that targets patients presenting within the ultra-early time window—prior to follow-up imaging—may prove more effective.

Nonetheless, early follow-up imaging remains clinically valuable. Beyond aiding prognostication, it facilitates early detection of complications, such as intraventricular hemorrhage, hydrocephalus, or mass effect, which may require prompt neurosurgical intervention (17). Moreover, the availability of ultra-early imaging data offers important insights into the pathophysiological dynamics of ICH, supporting the concept of non-linear growth with a rapid phase of HE early in the disease course. Concerns regarding radiation exposure should not preclude this approach, as most affected patients are elderly and present with a life-threatening condition; in addition, low-dose CT protocols for follow-up imaging may further mitigate radiation risks (18).

One of the key strengths of this study is the large sample size within a multicenter cohort. Additionally, ICH volumes were manually segmented on both admission and follow-up NCCT scans, with experienced readers and expert neuroradiologists reviewing the segmentations to ensure accuracy. The study also benefits from comprehensive clinical data collected over an extended period. Furthermore, the core analysis was based on a prospectively planned imaging workflow. In patients transported by the mobile stroke unit, an initial NCCT was routinely performed prehospital, followed by an ultra-early follow-up scan upon hospital arrival. This standardized protocol allowed a consistent and unbiased assessment of early hematoma dynamics under routine clinical conditions.

Certain limitations should be acknowledged. Although data collection in this study was prospectively designed and conducted, the ultra-early imaging cohort was relatively small, potentially limiting the generalizability of some findings. Nevertheless, despite its moderate size, the prospective cohort remains one of the largest reported to date in the ultra-early time window. Future studies with larger prospective datasets are warranted to confirm and extend our results. In addition, clot retraction and secondary injury processes, such as perihematomal edema formation, may have influenced the final hematoma volume; however, this effect was likely minimal in the prospective sub-study, where all imaging occurred within the first 24 h. Intraventricular hemorrhage was not included in our volume measurements, as mechanisms of hematoma expansion likely differ once blood extends into the ventricular system, where tissue resistance is minimal.

In summary, ultra-early NCCT follow-up appears to be a valuable tool for diagnosing hematoma expansion in acute ICH. Additional studies are needed to validate these findings and optimize the timing of diagnostic algorithms. For prevention of HE, early presentation remains a precondition for effective treatment and may represent the most promising approach for future RCTs aimed at improving patient outcomes.

## Data Availability

The original contributions presented in the study are included in the article, and further inquiries are directed to the corresponding author.
